# A Butyl Methacrylate Monolithic Column Prepared *In-Situ* on a Microfluidic Chip and its Applications

**DOI:** 10.3390/s90503437

**Published:** 2009-05-08

**Authors:** Yi Xu, Wenpin Zhang, Ping Zeng, Qiang Cao

**Affiliations:** National Key Disciplines Lab of Novel Micro-Nano Devices and System Technology, and International R&D Center of Micro-Nano Systems and New Materials Technology, and Microsystem Research Center, and College of Chemistry and Chemical Engineering, Chongqing University, Chongqing, 400044, China; E-mails: zhang wenpinchina@yahoo.com.cn (W.-P.Z.); zp860128@163.com (P. Z.); tianmao6971@163.com (Q. C.)

**Keywords:** BMA monolithic column, separation and preconcentration, microfluidic chip

## Abstract

A butyl methacrylate (BMA) monolithic column was polymerized *in-situ* with UV irradiation in an ultraviolet transparent PDMS micro-channel on a homemade micro-fluidic chip. Under the optimized conditions and using a typical polymerization mixture consisting of 75% porogenic solvents and 25% monomers, the BMA monolithic column was obtained as expected. The BET surface area ratio of the BMA monolithic column was 366 m^2^·g^-1^. The corresponding SEM images showed that the monolithic column material polymerized in a glass channel was composed of uniform pores and spherical particles with diameters ranging from 3 to 5 μm. The promethazine–luminal–potassium ferricyanide chemiluminescence system was selected for testing the capability of the column. A flow injection analytical technique–chemiluminescence (FIA–CL) system on the microfluidic chip with a BMA monolithic column pretreatment unit was established to determine promethazine. Trace promethazine was enriched by the BMA monolithic column, with more than a 10-fold average enrichment ratio. The proposed method has a linear response concentration range of 1.0×10^-8^ - 1.0×10^-6^g·mL^-1^ and the detection limit was 1.6×10^-9^g·mL^-1^.

## 1. Introduction

Sample pretreatment is one of the most important steps in an analytical process. Recently, micro-fluidic systems have been investigated extensively for biological and chemical analysis because miniaturization requires smaller samples and offers lower reagent consumption and costs and higher throughput and performance. However, the capability of microfluidic devices to efficiently handle complex samples and the integration of sample pretreatment on the same microfluidic chip will be essential to the successful application of these microfluidic systems. In the Micro Total Analysis Systems (μ-TAS) field much attention has been paid to sample pretreatment units integrated on microfluidic chips [1]. To date, solid media have shown special advantages for practical sample analysis, and some pretreatment methods involving solid media integrated into microfluidic chips have been investigated recently. Beads are currently used in many analytical systems, although incorporating them into chips is really difficult [2]. On the other hand, membranes are readily incorporated into micro-fluidic systems, but have limited applicability due to their small size [3]. Fabricating microstructures on chips is an approach to increasing the surface to volume ratio in a chip as well as allowing multiple devices to be designed into a chip. This approach has the advantage that fabrication of multiple supports requires only a little more effort than fabricating a single support. Finally, monoliths can provide for a solid medium within a channel and can be easily fabricated or modified to have a wide variety of functionalities [4].

Compared to traditional chromatography columns, the recently developed monolithic columns prepared *in-situ* have many advantages such as easy preparation and modification, low operational pressure, high resolution, large capacity, good permeability, fast mass transfer properties and good stability [5-7]. These monolithic columns can be used not only as stationary phases for capillary electro-chromatography and micro-column high performance liquid chromatography (μ-HPLC), but also as matrices for sample pretreatment and enzyme reactors. Due to their simplicity, speed and effectiveness, monoliths are especially suited for integration into microfluidic devices, so it is not surprising that monolithic columns have attracted considerable attention and have been applied widely in micro-fluidic chip analytical systems in recent years [8]. In this work a butyl methacrylate (BMA) monolithic column was polymerized *in-situ* by UV irradiation in a microchannel on a homemade microfluidic chip for use as a pretreatment device. The fabrication was accomplished successfully and the resulting device applied to preconcentrate trace promethazine in synthetic plasma samples. It was thus demonstrated that the butyl methacrylate monolithic column prepared *in-situ* was highly effective as a pretreatment unit on a microchip to separate and concentrate some practical samples.

## 2. Experimental Section

### 2.1. Chemicals and instruments

A multifunction chemiluminescence analysis system with a PMT detector (MCDR-A, Xi'an Remax Electronic Co., Ltd.) and syringe pump (Harvard Apparatus, Holliston, MA) was used. Ethylene dimethacrylate (EDMA) and polydimethylsiloxane (PDMS) were purchased from the Dow Corning Corporation (Midland, MI, U.S.A). Butyl methacrylate (BMA), 2,2′-azobis-(2-methylpropionitrile) (AIBN), potassium ferricyanide, luminol, ammonium acetate, formic acid, acetic acid, ethanol, methanol, acetone and acetonitrile were acquired from Chongqing Chuandong Chemical Engineering Reagent Company (Chongqing, P.R. China). γ-MAPS was purchased from the Shanghai Reagent Company (Shanghai, P.R. China). Promethazine hydrochloride was purchased from the Medicament Company (Beijing, P.R. China). All aqueous solutions were prepared using double distilled water.

Stock standard solution containing 1×10^-3^mol·L^-l^ of drug was prepared by dissolving a weighed amount of promethazine hydrochloride in ammonium acetate (pH=9.3). Standard solutions were prepared daily by appropriate dilution of the stock solution in ammonium acetate (pH=9.3). The promethazine stock solution was diluted in a freshly human serum sample to a concentration of 2.0×10^-9^ g·mL^-l^. A 1×10^-2^ mol·L^-l^ stock solution of potassium ferricyanide was prepared by dissolving the required amount of compound in distilled water. Diluted solutions of potassium ferricyanide were prepared by mixing portions of the stock solution with the required amounts of NaOH/NaHCO_3_ solution.

### 2.2. Microchip Fabrication

PDMS is useful for microfluidic fabrication and application because of several important properties, including its mechanical flexibility, gas permeability and optical transparency. Moreover, its elasticity and hermetic self-sealing properties make multilayer construction of PDMS-based devices relatively straightforward. PDMS molding is used almost exclusively for rapid prototyping in corporate environments because of its simplicity and fast turnaround time. In this paper, the microchips employed in the experiments were fabricated with PDMS and glass using standard techniques. The PDMS chip is cured, removed, and then pressed or bonded onto a glass substrate to create a complex microchannel by the monolithic pouring method. The bottom chip was fabricated in glass using conventional wet chemical etching. In a final step, a glass chip was bonded to the PDMS chip to yield a network of closed channels. The upper layer and the bottom layer having different channels. The upper layer had the preconcentration microchannels (AB, 5 mm length and 150 μm diameter) and the lower PDMS layer had “Y” shape reaction microchannels (150 μm wide and 150 μm deep). The reservoirs (R_1_, R_2_ R_3_ and R_4_) were punched on the upper PDMS layer using a round hollow punch,

### 2.3. Monolith column polymerization

For the polymerization of the monolith column butyl methacrylate (BMA), ethylene dimethacrylate (EDMA), 2,2′-azobis-(2-methylpropionitrile) and methanol/ethanol were used as the monomer, cross-linking agent, initiator and porogenic solvents, respectively. The detailed monolith column polymerization processes was carried out as follows: first, the microchannels flushed by sodium hydroxide solution and double distilled water were washed with silanization solution containing 30% (V/V) γ-MAPS in acetone. Prior to mixing with the porogenic solvents, EDMA and BMA were mixed with fresh basic alumina powder to remove the added inhibitor. After purging with nitrogen for 3 min, the mixture consisting of 75% porogenic solvents and 25% monomers were pumped into microchannel for the subsequent polymerization. The porogenic solvent used was a mixture of methanol and ethanol with a ratio of 5:3. A 1:1 mixture of BMA and EDMA was used as monomer. Then, 365 nm UV light was used to irradiate the mixture from the top from a 5 cm distance through a mask to control the location of the BMA monolithic column on the microchip. The BMA monolithic column was polymerized *in-situ* in an ultraviolet transparent PDMS microchannel on a homemade microfluidic chip; it was finally washed with methanol at 2.0 μL·min^-1^ for 30 min. The monolithic column was then attached to the FIA-CL micro-syringe pump to separate and preconcentrate trace promethazine in the synthetic plasma samples.

### 2.4. Sample analysis process by microfluidic chip

#### 2.4.1. Sample analysis system

**Figure 1.**
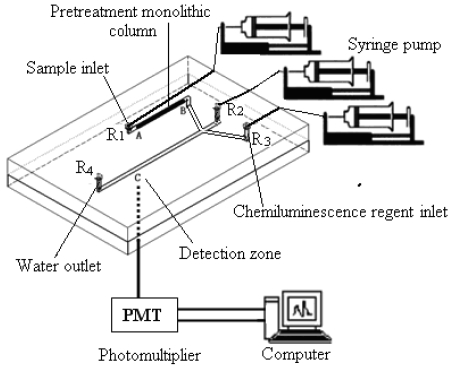
Schematic diagram of the FIA-CL system based on the homemade microfluidic chip with monolithic column

The schematic diagram of the microfluidic chip analytical system is shown in Figure 1. The whole analysis system consist of microfluidic chip, monolithic column, photomultiplier, computer and syringe pump. In this system, the reservoirs R_1_, R_2_ and R_3_ were connected to microsyringe pumps, R_1_ for sample solution while R_2_ and R_3_ for chemiluminescence reagent and R_4_ for waste water. In such configuration, a sample was enriched by the pretreatment monolithic column when the sample was loaded to the microchannels in the microchip with a microsyringe pump. The enriched sample was washed with a suitable solution, and then the eluted solutions were injected into the luminol mixture solution. All the solutions were mixed in a microchannel just before the detection zone in the homemade microfluidic chip. The intensity of the emission response was obtained in this way by photomultiplier tube (PMT) detector in a dark housing.

#### 2.4.2. Sample loading and elution

The BMA monolithic column on the microchip was washed and preconditioned as follows: after washing with acetonitrile for 10 min at 3.0 μL·min^-1^, the monolithic column was preconditioned with 10 mM ammonium acetate buffer (pH 9.3) for 10 min at the same flow rate. The promethazine stock solution was diluted in ammonium acetate (pH = 9.3) to a final concentration of 2×10^-9^ g·mL^-1^, and a volume of 30 μL of the sample was loaded to the BMA monolithic column at a flow rate of 0.2 μL·min^-1.^ After washing the column with ammonium acetate (pH = 9.3) at a flow rate of 2.0 μL·min^-1^ for 10 min, the retained promethazine was eluted with acetonitrile containing 0.1% formic acid, which was pumped into the reaction channel in the glass layer at a flow rate of 0.5 mL·min^-1^.The promethazine eluted solutions were injected into the mixture of luminol solution (1.0×10^-4^ mol·L^-1^) and potassium ferricyanide (2.5×10^-4^ mol·L^-1^) under basic conditions of NaOH/NaHCO_3_ (C_NaOH_:C_NaHCO3_ = 7:5). The CL intensity was measured by photomultiplier tube. The methodology of promethazine detection was developed by the chemiluminescence reaction of promethazine with luminol and ferricyanide in basic medium.

## 3. Results and Discussion

### 3.1. Microchip Fabrication technology

**Figure 2.**
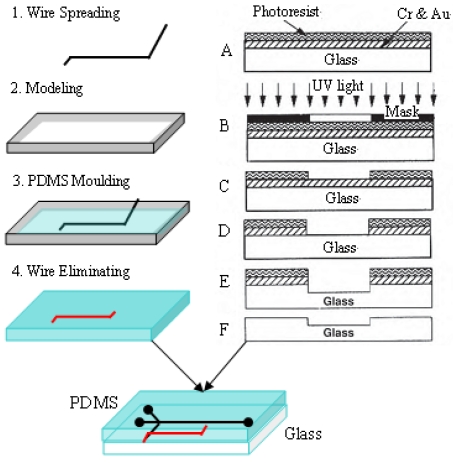
Process flow for creating PDMS microfluidics by cast molding technique (left) and stepwise illustration of wet chemical etch process for etching glass chip (right).

In PDMS fabrication technology, the process of creating microfluidic channels in PDMS using SU-8 as a mold is complicated and very difficult to carry out in an ordinary laboratory. On the other hand, the monolithic PDMS pouring method offers simpler fabrication procedures, lower costs, faster turnaround time and higher integration and mechanical performance etc; in addition, the integrated microchannel can be used repeatedly and it avoids the problem of leakage, which make it useful for biological and chemical analysis in a microfluidic chip analysis system. In this work, highly hydrophobic and surface polished copper/platinum wire was used as the channel moulding material in order to minimize the channel distortion and surface roughness. The size of microfluidic channels could be controlled by simply changing the size of the copper/platinum wire used. With all this in mind, the PDMS microchips used in the experiment were fabricated by the monolithic pouring method as follows: the PDMS oligomer and crosslinking prepolymer of PDMS agent is mixed in a mass ratio of 10:1. A convenient way of mixing these agents is to place both in a disposable plastic bag, heat seal, and mix manually. The mixture can be placed under vacuum for degassing. The PDMS mixture is poured onto the homemade wire shaped mold and cured for 4 h at 60°C (see Figure 2.3). After cooling, the PDMS can be carefully peeled off the mold (see Figure 2.4). Debris on the PDMS surface must be removed after punching the holes because the presence of small particles can be detrimental to the bonding of PDMS to glass substrate. This process yields a PDMS polymer sheet with whole microchannels. A schematic of the microchip described in this paper is shown in Figure 2. The length of channels is 20 mm, and the diameter of the channel was 100 μm. The bottom chip was fabricated in glass using conventional wet chemical etching as described in Figure 2. The detailed microfluidic channel fabrication process was as follows: firstly, a photomask is required to prepare the microchannel by computer aided design (CAD) program. A glass substrate with a chrome-gold layer and photoresist coating is shown in Figure 2.A. A metal layer comprised of chromium and gold is vapor deposited under vacuum on the surface of the glass. Then, a positive photoresist was coated on the metal surface using a photoresist coater developer at 3,500 rpm. The position master mask on the surface of the photoresist is created and the photoresist exposed using ultraviolet light (see Figure 2.B). After the photoresist has been developed, mask features are defined on the chrome-gold layer (see Figure 2.D). The metal layer has been etched in the desired pattern, leaving glass exposed in this pattern. The glass has been etched to the proper depth (see Figure 2.E). The photoresist and metal layers have been removed (see Figure 2.F). Micro channels with 150 μm width and 150 μm depth were fabricated on one glass substrate in this way. In a final step, the PDMS film is sealed to the flat glass surface to complete the microfluidics system. The PDMS can be sealed with manual pressure to create fluidic devices that do not need to withstand high pressures. If these are immediately pressed together, a tight bond will be created. The whole microchannel network is illustrated in Figure 1.

### 3.2. Structure and performance of the in-situ polymerized monolith column

**Figure 3.**
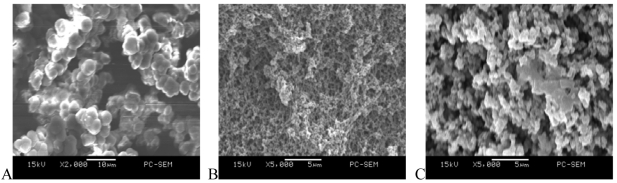
SEM images of BMA monolithic columns with different porogenic solvents under UV initiation. (A) methanol–ethanol; (B) 1,4-butanediol–*n*-propanol; (C) 1,4-butanediol–n-propanol–de-ionized water

With the polymerization procedure described in the Experimental section, a BMA monolithic column could be reproducibly formed in the microchannel. Compared with thermal polymerization, the polymerization with UV irradiation can provide a simple, rapid and efficient method for polymer preparation *in-situ* in a microfluidic chip. The polymerization conditions, such as initiation approach, polymerization time, the ratio of monomers and porogenic solvents, were investigated in detail. The structure and performance of the *in-situ* polymerized monolith columns formed with polymerization times under UV irradiation conditions ranging from 1 h to 10 h were tested. The results indicated that the monolith column with the best features was formed when the polymerization time was 4 h. Under the mentioned conditions, the composition and ratio of porogenic solvents, including 1,4-butanediol – *n*-propanol, 1,4-butanediol – *n*-propanol – de-ionized water and methanol – ethanol were investigated in detail. A mixture of BMA and EDMA with a ratio of 1 to 1 was used as monomer. The prepared BMA monolithic column was polymerized from a typical polymerization mixture consisting of 75% methanol – ethanol porogenic solvents and 25% monomers. The SEM images of the monolith column prepared *in-situ* in a PDMS microchannel with different porogenic solvents are shown in Figure 3. The results demonstrated that a mixture of methanol and ethanol with a ratio of 5 to 3 (*v:v*) was the optimum porogenic solvent. Under the optimized conditions, the prepared BMA monolithic column was obtained as predicted and was characterized using BET Surface Area Analyzer, Infrared Spectroscopy and Scanning Electron Microscopy (SEM). The ratio of BET surface area of the BMA monolithic column was 366 m^2^·g^-1^. The SEM images (see Figure 3.A) showed that the monolithic column material polymerized in a glass channel was composed of uniform pores and spherical particles with diameters ranging from 3 μm to 5 μm. Other experiments also showed that the BMA monolithic column also had good mechanical properties.

### 3.3. Microfluidic chip analytical system

An analysis system based on a polydimethylsiloxane (PDMS) microfluidic chip with a monolithic column was successfully fabricated and used to preconcentrate trace promethazine in synthetic serum samples. The processes, consisting of sample pretreatment, chemiluminescence reaction and signal detection procedure could be performed completely on the homemade microfluidic chips. The micro- fluidic chip analytical system could be not only increase the applicability monolithic columns in Micro Total Analysis Systems, but also provides a simple miniaturized microfluidic device for medical analysis, environmental monitoring, biochemical analysis, and microchemistry.

Moreover, owing to its ultraviolet transparency characteristics, is was easy to achieve monolithic column polymerization in the integrated microchannel on the PDMS microchip under UV irradiation. The location of the polymer materials was controlled by a low-resolution mask with the desired length of open window made from attached aluminum foil. A portable 365 nm lamp was used to shine UV light from the bottom through the mask onto the microchip. The distance between the light and the device was 5 cm. After polymerization for 4 h, the solution was kept in the channel for 5 min to reduce the number of unreacted free radicals. The performance of pretreatment monolithic column polymerization was affected by the power of UV light and the thickness of PDMS which can absorb the energy of UV irradiation. In such configuration, BMA monolithic column was polymerized successfully in the homemade microfluidic chip, and the integrated microfluidic chip analytical system was established.

### 3.4. Promethazine detection by FIA-CL on the microchip

**Figure 4.**
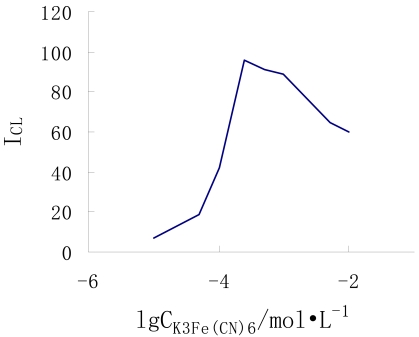
Effect of potassium ferricyanide concentration on CL intensity

**Figure 5.**
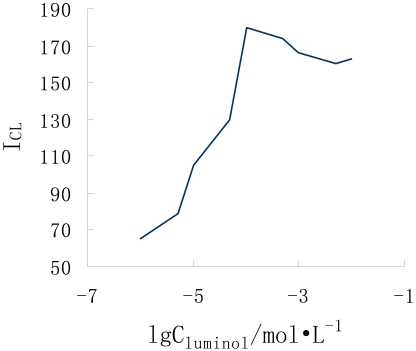
Effect of luminol concentration on CL intensity

In order to test the compatibility of the monolithic column in the microchip analytical system, the promethazine-luminol-ferricyanide chemiluminescence system was selected to test the capability of the BMA monolithic column combined with the flow injection analytical technique (FIA). The chemiluminescense reaction conditions were optimized by the univariate approach. The influence of the reaction medium was tested by varying the concentrations of the different reagents used. The concentration ratio of NaOH/NaHCO_3_ buffer solution was varied over a concentration range from 1:10 to 10:1. The results indicated that the ratio 7:5 gave the greatest sensitivity. The concentration of oxidant was determined to have a significant effect on the emission intensity at 3 μL·min^-1^. Concentration ratios of luminol and potassium ferricyanide ranging from 1.0×10^-5^ mol·L^-1^ to 1.0×10^-2^ mol·L^-1^ were examined. By increasing luminol concentration up to 1.0×10^-4^mol·L^-1^ and potassium ferricyanide concentration up to 2.5×10^-4^ mol·L^-1^ the emission intensity increased as shown in Figures 4 and 5. Above this concentration the emission intensity decreased, probably due to the absorbing of the emitted light by the the potassium ferricyanide, so 1.0×10^-4^ mol·L^-1^luminol and 2.5×10^-4^ mol·L^-1^ potassium ferricyanide were considered to be optimum.

### 3.5. Sample analysis

The promethazine stock solution was diluted in a freshly collected human serum sample to a concentration of 2.0×10^-9^ g·mL^-l^. The washing and preconditioning steps were as described in Section 2.4.2. In the microfluidic chip FIA-CL analysis system, the promethazine was enriched by the BMA monolithic column when a volume of 15 μL of the human serum sample was loaded to the microchannels in the microchip at a flow rate of 0.5 μL·min^-1^.

**Figure 6.**
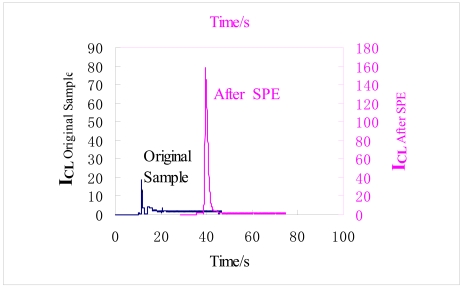
The results of promethazine in the synthetic sample by the microfluidic chip FIA-CL system.

The promethazine enriched column was washed with ammonium acetate buffer solution, and then the solutions eluted with acetonitrile containing 0.1% formic acid were injected into the mixture of luminol solution and potassium ferricyanide under basic conditions of NaOH/NaHCO_3_. The intensity of the emission response was obtained as shown in Figure 6, which indicates that an average enrichment ratio above 10 times could be achieved under optimized conditions. The proposed method has been successfully applied to the measurement of promethazine in the synthetic plasma samples, where it has been shown that the linear response concentration range was 1.0×10^-8^∼1.0×10^-6^g·mL^-1^, and the detection limit was 1.6×10^-9^ g·mL^-1^.

## 4. Conclusions

Research in integrated microfluidic devices has expanded to include sample preparation, fluid handling, microreactors and separation systems etc, which have been applied in medical analysis, environmental monitoring, biochemical analysis, and microchemistry. Based on the problem of pretreatment in complex samples, this paper have demonstrated that microfluidic chip devices with integrated monolithic column pretreatment units can carry out sequential test steps consisting of sample preparation, chemical reaction and detection for selected samples. The experimental results show that a butyl methacrylate monolithic column could be polymerized *in-situ* with UV irradiation in an ultraviolet transparent PDMS microchannel on a homemade micro-fluidic chip. The pretreatment devices with a monolith pretreatment unit was successfully applied to preconcentrate trace promethazine in synthetic plasma samples, and it was thus demonstrated that SPE monolithic column pretreatment units on a microchip show great potential for biochemical analysis, especially in the measurement of trace medicines in plasma samples.

## References

[1] Yang Y., Li C., Kameoka. J., Lee K.H., Craighead H.G. (2005). A polymeric microchip with integrated tips and *in-situ* polymerized monolith for electro-spray mass spectrometry. Lab Chip..

[2] Verpoorte E. (2003). Beads and chips: new recipes for analysis. Lab Chip..

[3] Wang P.C., DeVoe D.L., Lee C.S. (2001). Integration of polymeric membranes with microfluidic networks for bioanalytical applications. Electrophoresis.

[4] Svec F., Tennikova T.B., Deyl Z. (2003). Monolithic Material: Preparation, Properties and Applications.

[5] Tan A., Benetton S., Henion J. D. (2003). Chip-Based Solid-Phase Extraction Pretreatment for Direct Electro-spray Mass Spectrometry Analysis Using an Array of Monolithic Columns in a Polymeric Substrate. Anal. Chem..

[6] Throckmorton D., Shepodd T., Singh A. (2002). Electro-chromatography in microchips: reversed-phase separation of peptides and amino acids using photo-patterned rigid polymer monoliths. Anal. Chem..

[7] Ping G.C., Yuan X.L., Zhang Y.K. (2001). Preparation and Application of Monolithic Column. Chin. J. Anal. Chem. H..

[8] Wei F., Lin B., Feng Y.Q. (2007). Applications of Monoliths in Sample Preconcentration. Chin. J. Chromatogr. H..

